# The development and immunosuppressive functions of CD4^+^ CD25^+^ FoxP3^+^ regulatory T cells are under influence of the adenosine-A2A adenosine receptor pathway

**DOI:** 10.3389/fimmu.2012.00190

**Published:** 2012-07-05

**Authors:** Akio Ohta, Radhika Kini, Akiko Ohta, Meenakshi Subramanian, Manasa Madasu, Michail Sitkovsky

**Affiliations:** New England Inflammation and Tissue Protection Institute, Northeastern University, BostonMA, USA

**Keywords:** regulatory T cells, adenosine, immunosuppression, A2A adenosine receptor, cancer, autoimmune, transplantation

## Abstract

The A2A adenosine receptor (A2AR)-mediated immunosuppression is firmly implicated in the life-saving down-regulation of collateral tissue damage during the anti-pathogen immune response and in highly undesirable protection of cancerous tissues during anti-tumor immune response. Therefore, depending on specific clinical situation there is a need to either weaken or strengthen the intensity of A2AR signal. While the A2AR-mediated immunosuppression was shown to be T cell autonomous in studies of effector T cells, it was not clear how A2AR stimulation affects regulatory T cells (Treg). Here we show in parallel assays that while A2AR stimulation on T cells directly inhibits their activation, there is also indirect and longer-lasting T cell inhibitory effect through modulation of Treg. A2AR stimulation expanded CD4^+^ CD25^hi^ FoxP3^+^ cells, which also express CD39, CD73, and CTLA-4. Treg cultured with A2AR agonist showed increased expression of CTLA-4 and stronger immunosuppressive activity. There was a significant increase of Treg cell number after A2AR stimulation. The CD4^+^ FoxP3^+^ population contained those induced from CD4^+^ CD25^−^ cells, but CD4^+^ FoxP3^+^ cells predominantly derived from CD4^+^ CD25^+^ natural Treg. Thus, A2AR stimulation numerically and functionally enhanced Treg-mediated immunosuppressive mechanism. These data suggest that the A2AR-mediated stimulation of lymphocytes using A2AR agonists should be considered in protocols for *ex vivo* expansion of Treg before the transfer to patients in different medical applications.

## Introduction

It is now no longer controversial and it is now widely accepted that there are professionally immunosuppressive regulatory T cells (Treg), which have been first identified and characterized by observations of autoimmunity in mice depleted of CD4^+^ CD25^+^ T cell subpopulation (Sakaguchi et al., 1982, 1985). The mechanisms of development and immunoregulatory functions of Treg have been subjects of extensive investigation (Lu and Rudensky, 2009; Ohkura and Sakaguchi, 2011; Rudensky, 2011; Sakaguchi, 2011). Treg are also of great interest due to their potential to treat immunological diseases and control physiological and pathological immune responses. However, there are still important and yet to be answered questions about the influence of the microenvironments in lymphoid and inflamed tissues in the development and immunoregulatory functions of Treg.

Here we investigated modulation of Treg-dependent immunosuppressive activities by the adenosine-A2AR signaling which was shown to represent the powerful physiological immunosuppressive mechanism (Ohta and Sitkovsky, 2001; Lukashev et al., 2004; Sitkovsky et al., 2004; Belikoff et al., 2011) that protects both normal (Ohta and Sitkovsky, 2001; Thiel et al., 2005; Ohta et al., 2007) and cancerous tissues (Ohta et al., 2006) from inflammatory damage. It is believed that the adenosine-A2AR pathway has evolved as a negative feed-back immunosuppressive mechanism that limits the extent of the collateral tissue damage by activated immune cells during anti-pathogen responses (Sitkovsky and Lukashev, 2005; Sitkovsky and Ohta, 2005). This mechanism may regulate the other major, but evolutionary younger immunosuppressive mechanisms including Treg (Pouliot et al., 2002; Cadieux et al., 2005; Sitkovsky, 2009).

But is there really a relation between Treg and immunosuppressive effect of extracellular adenosine? There are several lines of converging suggestive evidence that Treg activity is mediated by the accumulation of extracellular adenosine. The extracellular adenosine was first implicated in Treg activity during the unbiased screening of differential expression of surface antigens on Treg revealing that Treg express high levels of the extracellular adenosine-generating enzymes CD73 ecto-enzyme, an 5′-nucleotidase (Kobie et al., 2006) and the upstream ecto-enzyme CD39 apyrase (ecto ATPase/ADPase; Deaglio et al., 2007). These studies suggested that the CD39 and CD73 ecto-enzymes on Treg play a role in immunosuppressive loops generating extracellular adenosine that down-regulates T cell activation (Whiteside, 2012).

However, there is still paucity of data that may firmly implicate the adenosine and A2AR in functions of Treg. It was suggested that the A2AR stimulation might promote the induction of adaptive regulatory T cells, but this claim is lacking direct evidence. The ability of A2AR agonist to increase the expression of foxP3 mRNA was studied (Zarek et al., 2008), but effects of A2AR agonist on the number and immunosuppressive activity of Treg are not known.

In this study, we provide evidence that the engagement of A2AR results in expansion of Treg and promotes immunoregulatory activity of Treg. These data support the overall model of the adenosinergic regulation of Treg functions (Sitkovsky, 2009).

## Materials and methods

### Mice

C57BL/6 (Thy1.2^+^) and BALB/c mice were purchased from Charles River Laboratories (Wilmington, MA). B6.PL-Thy1a/CyJ mice (Thy1.1^+^ C57BL/6 mice) were purchased from Jackson Laboratory. A2AR^−/−^ mice were backcrossed for 12 times to C57BL/6 mice (Chen et al., 1999). Mice were used at 8–12 weeks of age. The experiments were approved by the Northeastern University Institutional Animal Care and Use Committee and were carried out in accordance with the institutional animal care guidelines.

### Mixed lymphocyte culture (MLC)

Spleen cells from C57BL/6 mice (responder; H-2^b^) were stimulated with allogenic spleen cells. As stimulators, spleen cells from BALB/c mice (H-2^d^) were pretreated with mitomycin C (Sigma, St. Louis, MO). Responders (6 × 10^6^ cells) and stimulators (2 × 10^6^ cells) were cultured for 5 days in the presence or absence of A2AR agonist, 1 μM CGS21680 (CGS) or 1 μM 5′-N-ethylcarboxamidoadenosine (NECA). A2AR antagonist ZM241385 (ZM) was added at 1 μM to some samples. The concentrations of compounds are optimal to stimulate or antagonize A2AR according to our previous study (Ohta et al., 2009). The activated cells were restimulated with mitomycin C-treated BALB/c spleen cells for 2 more days in the same condition. NECA was obtained from Sigma (St. Louis, MO). CGS and ZM were from Tocris (Ellisville, MO).

### Flowcytometry

The resulted cells after MLC were analyzed by flowcytometry. Following antibodies were used to label surface molecules: PE-conjugated anti-CD4, anti-CD25, anti-CD39, anti-CD73, and FITC-conjugated anti-CD8, anti-H-2K^b^ and allophycocyanin (APC)-conjugated anti-CD4 antibodies. For the analysis of Treg, the cells were subsequently fixed and permeabilized using FoxP3 staining buffer set (eBioscience, San Diego, CA), and were labeled with APC-conjugated anti-FoxP3 and PE-conjugated anti-CTLA-4 antibodies. All antibodies were from BD Biosciences (San Diego, CA) except for anti-FoxP3, anti-CD39 (eBioscience) and anti-CD25 (Miltenyi Biotec, Auburn, CA) antibodies. The data were acquired using FACSCalibur (BD Biosciences).

### MLC in the absence of CD8^+^ cells

To enrich Treg after the culture, MLC was set up using CD8^+^-depleted C57BL/6 spleen cells. CD8^+^ cells were labeled with FITC-conjugated anti-CD8 mAb (BD Biosciences) and anti-FITC microbeads (Miltenyi Biotech) and removed using AutoMACS separator (Miltenyi Biotec). These responder cells were cultured with mitomycin C-treated stimulator cells as described above.

### Cell proliferation assay using CFSE-labeled cells

The extent of T cell proliferation was monitored by the stepwise dilution of fluorescence in CFSE-labeled cells. To label with CFSE (Molecular Probes, Eugene, OR), cells were washed with PBS and incubated with 1 μM CFSE for 8 min. To remove excess CFSE, the cells were washed twice with fetal calf serum.

### Regulatory activity of Treg

After MLC using CD8^+^-depleted responders for 7 days (2 days after restimulation), the regulatory activity was evaluated according to the inhibition of effector T cell proliferation. CD8^+^-depleted spleen cells from Thy1.1-expressing C57BL/6 mouse were labeled with CFSE and used as the source of responder T cells (Tresp). Tresp (2.5 × 10^4^ CD4^+^ cells) were co-cultured with the product of MLC, which contains Treg, so that the ratio of CD4^+^ cells in Tresp and CD4^+^ FoxP3^+^ cells in the MLC would be constant between groups. Tresp cell proliferation was induced with anti-CD3 mAb (0.1 μg/ml 145-2C11; BD Biosciences) for 2 days in a round-bottomed 96-well plate, and the extent of Tresp proliferation was analyzed after gating for Thy1.1^+^ CD4^+^ cells.

### Treg from CD4^+^ CD25^−^ cells

To start MLC in the absence of natural Treg, CD25^+^ cells were removed from the responder cells prior to the culture. Spleen cells were labeled with PE-conjugated anti-CD25 and anti-CD8 mAbs, and the labeled cells were depleted using anti-PE microbeads (Miltenyi Biotec) and AutoMACS. After 7-days MLC as described above, the appearance of CD4^+^ FoxP3^+^ cells was tested by flowcytometry.

### Natural Treg

CD4^+^ CD25^+^ cells were purified from spleen cells of Thy1.1-expressing C57BL/6 mice as described (Nagahama et al., 2007). CD24^+^ cells and CD8^+^ cells were removed from the spleen cells using FITC-conjugated antibodies and anti-FITC microbeads. Subsequently, CD25^+^ cells were retrieved by positive selection using PE-conjugated anti-CD25 antibody and anti-PE microbeads. This procedure achieves 95–98% pure CD4^+^ CD25^+^ cells. Responder cells of MLC were reconstituted by mixing Thy1.1^+^ CD4^+^ CD25^+^ cells (6 × 10^4^) with Thy1.2^+^ spleen cells depleted of CD8^+^ and CD25^+^ cells (3 × 10^6^). After 7-days MLC as described above, the origin of CD4^+^ FoxP3^+^ cells was separately analyzed for natural Treg-derived Thy1.1^+^ cells and CD4^+^ CD25^−^ cells-derived Thy1.1^−^ cells.

### cAMP induction in Treg

Purified CD4^+^ CD25^+^ cells (1.6 × 10^5^) were incubated with NECA or CGS for 15 min at 37°C. The concentration of A2AR agonists was 10 μM, and 1 μM for A2AR antagonist, ZM241385. cAMP levels were determined by ELISA (GE Healthcare, Buckinghamshire, UK).

### Statistics

Data represent mean ± SD. Statistical calculations were performed using Student's *t*-test. Statistical significance was accepted for *p* values less than 0.05.

## Results

**Figure 1:**
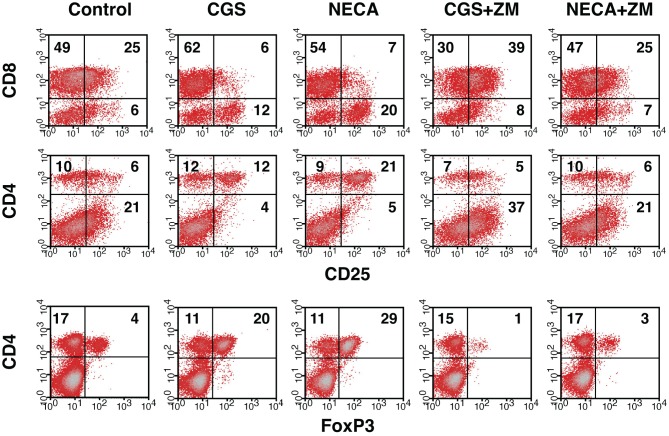
**Increase of Treg population by the stimulation of A2AR.** Mixed lymphocyte culture (MLC) was set up in the presence of A2AR agonist, CGS21680 (1 μM), or NECA (1 μM). After 5 days, the cultured cells were restimulated with the same allogenic stimulator cells for 2 more days in the same condition. A2AR stimulation inhibited CD25 expression in CD8^+^ cells (top row), whereas the population of CD4^+^ CD25^+^ cells was rather increased in the same culture (middle row). The change in CD4^+^ CD25^+^ cells correlated well with an increase of FoxP3-expressing CD4^+^ cells (bottom row). The addition of A2AR antagonist ZM241385 (1 μM) reversed the changes. Numbers in the panels represent percentages in each quadrant. The data shown here represent four separate experiments with similar results.

**Figure 2:**
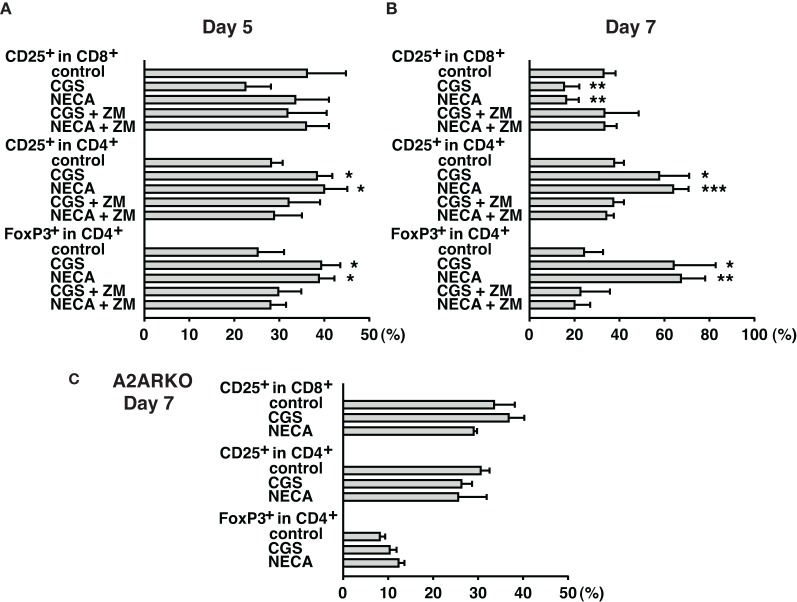
**Time-dependent changes of Treg increase during MLC with A2AR agonist.** Cell culture was done as described in Figure 1. Spleen cells from wild-type **(A,B)** and A2AR^−/−^ mice **(C)** were used as responder cells. Cells were analyzed by flowcytometry on day 5 **(A)**, and day 7 **(B,C)**. A2AR agonists inhibited CD8^+^ T cell activation and enhanced CD25/FoxP3 expression in CD4^+^ cells from wild-type mice, but not A2AR^−/−^ mice. Data represent average ± SD of 3–4 separate experiments. ^*^P < 0.05; ^**^P < 0.01; ^***^P < 0.001 vs. control MLC.

Immunosuppressive effects of extracellular adenosine are at least in part due to the inhibition of T cell activation. We have shown that stimulation of A2AR inhibits activation of effector T cells and their effector functions (Ohta et al., 2009). In agreement with our previous studies, A2AR agonists, CGS21680 (CGS) and NECA, blocked upregulation of CD25 on CD8^+^ T cells during MLC suggesting impaired activation of the effector T cells in response to allogenic stimulation (Figure 1 top panels). Interestingly, however, the proportion of CD25^+^ CD8^−^ cells was found to rather increase when CGS or NECA was added to the culture. This prominent increase of CD25^+^ cells by A2AR stimulation belonged to CD4^+^ population (Figure 1 middle panels). Most CD4^+^ CD25^+^ cells after treatment with CGS and NECA were distinct in their higher expression of CD25. Since A2AR stimulation is generally immunosuppressive, the increase of CD4^+^ CD25^+^ cells was not likely to represent activation of CD4^+^ effector T cells. Indeed, massive increase of FoxP3^+^ cells suggested that what appeared as CD4^+^ CD25^hi^ cells after A2AR stimulation could be regulatory T cells (Figure 1 bottom panels). Statistically significant changes were observed on day 5 of MLC and became more prominent on day 7 (Figures 2A,B). The decrease of CD8^+^ CD25^+^ cells and the increases of CD25^+^ and FoxP3^+^ proportions in CD4^+^ cells by the addition of CGS and NECA were all blocked by A2AR antagonist ZM241385 (Figures 1 and 2). A2AR-dependence of these changes was also confirmed by experiments using A2AR^−/−^ responder cells in which CGS and NECA failed to block CD8^+^ cell activation and to induce CD25 and FoxP3-expressing CD4^+^ cells (Figure 2C).

**Figure 3:**
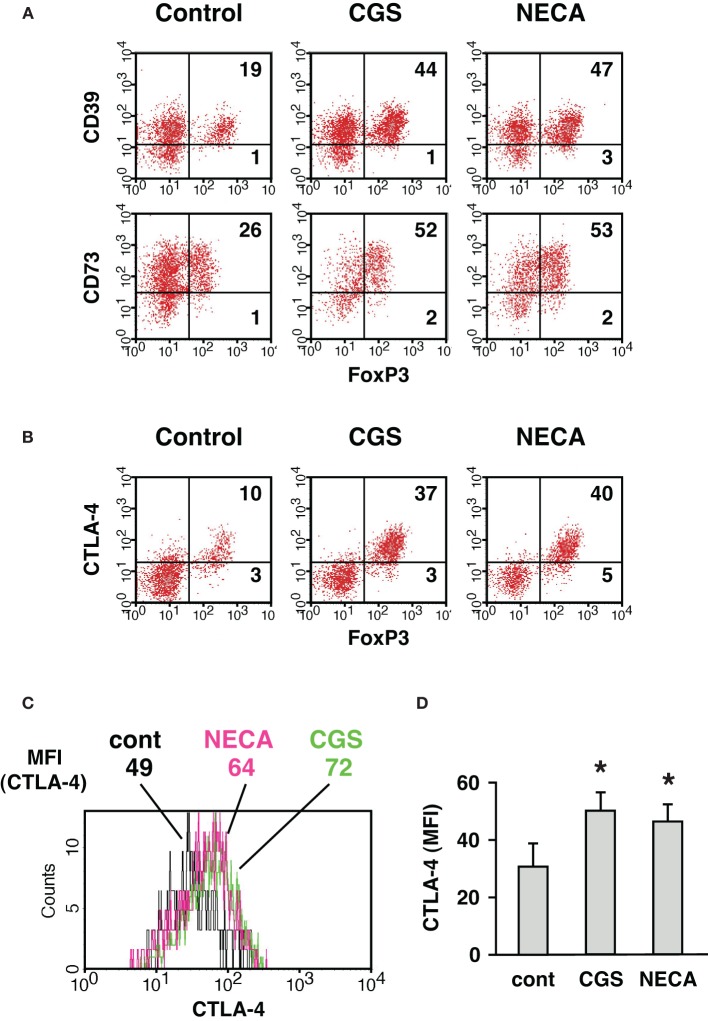
**Increase of CD4^+^ cells expressing CD39, CD73, and CTLA-4 when cultured in the presence of A2AR agonist.** MLC was done as described in Figure 1. **(A)** CD39 and CD73 expression in CD4^+^ FoxP3^+^ cells. The data was gated for H-2K^b+^ CD4^+^ cells. **(B)** CTLA-4 expression was analyzed by intracellular staining together with FoxP3. The data was gated for H-2K^b+^ CD4^+^ cells. Numbers in the panels represent percentages in each quadrant. **(C)** Histogram plots of CTLA-4 intensity in CD4^+^ FoxP3^+^ cells. Numbers represent mean fluorescence intensity (MFI) of CTLA-4. The data shown here represent five separate experiments with similar results. **(D)** Statistically significant increase of CTLA-4 levels by treatment with A2AR agonists. Data represent average ± SD of five separate experiments. ^*^P < 0.05 vs. control MLC.

We further characterized A2AR-mediated increase of CD4^+^ CD25^+^ population. The increased CD4^+^ cells expressed not only CD25 and FoxP3 but also CD39, CD73 (Figure 3A) and CTLA-4 (Figure 3B), which are closely relevant to immunoregulatory activity of Treg (Kobie et al., 2006; Deaglio et al., 2007; Sakaguchi et al., 2009). These results further implied that the emerging CD4^+^ CD25^+^ cells in the culture with A2AR agonists were Treg. Moreover, MLC in the presence of CGS or NECA upregulated CTLA-4 levels in CD4^+^ FoxP3^+^ cells (Figures 3C,D). CTLA-4 is constitutively expressed in Treg and plays an important role in the regulatory activity (Wing et al., 2008; Sakaguchi et al., 2009; Pandiyan et al., 2011). Therefore, it was also speculated that A2AR stimulation might induce CD4^+^ cells having enhanced regulatory activity.

**Figure 4:**
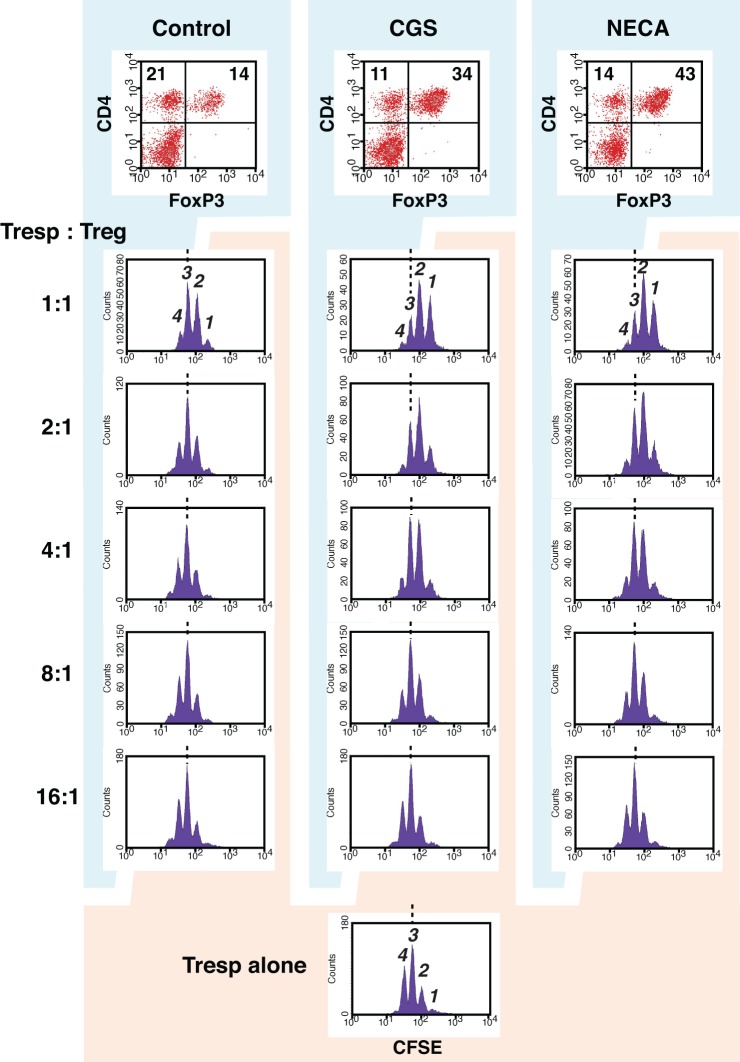
**A2AR stimulation promoted regulatory activity of Treg.** To enrich Treg in MLC, CD8^+^ cells were depleted from responder cells prior to the culture. It was confirmed that the treatment with CGS and NECA increased Treg population in this culture condition (top panels). The regulatory activity on T cell proliferation was determined by CFSE assay. CD8^+^-depleted spleen cells from Thy1.1-expressing C57BL/6 mouse were labeled with CFSE and used as responder cells (Tresp). Tresp containing 2.5 × 10^4^ CD4^+^ cells were co-cultured with the product of MLC, which contains Treg. Tresp:Treg in the figure is the ratio of CD4^+^ cells in Tresp to CD4^+^ FoxP3^+^ cells in the MLC. The extent of CD4^+^ Tresp cell proliferation was analyzed 2 days after the stimulation with anti-CD3 mAb. The histograms were gated for Thy1.1^+^ CD4^+^ cells. Broken lines indicate the same peak (peak 3). The data shown here represent three separate experiments with similar results.

**Figure 5:**
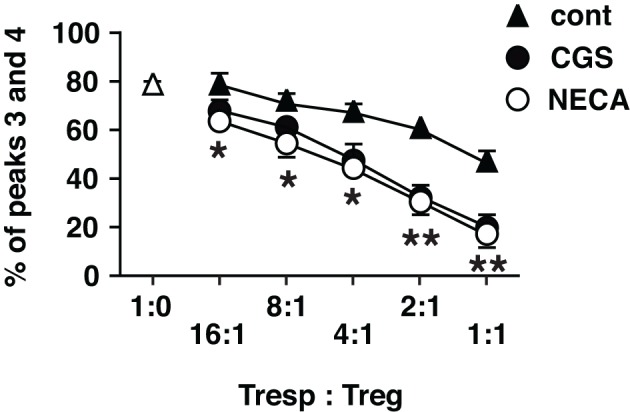
**Statistically significant enhancement of T cell inhibitory activity of Treg cultured with A2AR agonists.** The data of CFSE assay was presented as proportion of cells which entered into extensive proliferation. Numbers represent combined percentages of peaks 3 and 4 in Figure 4. Data represent average ± SD of three separate experiments. ^*^P < 0.01; ^**^P < 0.001 for both control vs. CGS and control vs. NECA.

Accordingly, we sought to examine regulatory activity of these cells. Since activated CD8^+^ T cells are predominant in regular MLC, which is inconvenient as the source of Treg in the assay of regulatory activity, we set up MLC after depletion of CD8^+^ responder cells. In this CD4^+^ MLC, the proportion of FoxP3^+^ cells was confirmed to increase by A2AR agonists (Figure 4 top panels). Immunoregulatory activity of these CD4^+^ FoxP3^+^ cells was evaluated by the inhibitory effect on T cell proliferation. The assay was normalized so that the same number of CD4^+^ FoxP3^+^ cells would be added to the constant number of CFSE-labeled CD4^+^ T cells. Comparing to the uninhibited control (Tresp alone), the addition of CD4^+^ FoxP3^+^ cells dose-dependently inhibited T cell proliferation. When the product of control MLC was added at 2:1 (Tresp: Treg), these Treg caused modest decrease of responder T cell proliferation (Figure 4 left). Such degree of T cell inhibition, however, was observed when the product of CGS or NECA-treated MLC was added at 8:1 (Figure 4 middle and right, Figure 5). Similarly, a larger number of control Treg (1:1) caused more significant reduction of proliferation, while this pattern corresponded to the result with CGS or NECA-treated MLC at 4:1 (Figures 4 and 5). A higher number of CGS or NECA-treated MLC product (2:1 and 1:1) inhibited T cell proliferation even stronger. This result confirmed that A2AR stimulation resulted in emergence of Treg and their regulatory activity was approximately 4-times stronger than that of control Treg.

**Figure 6:**
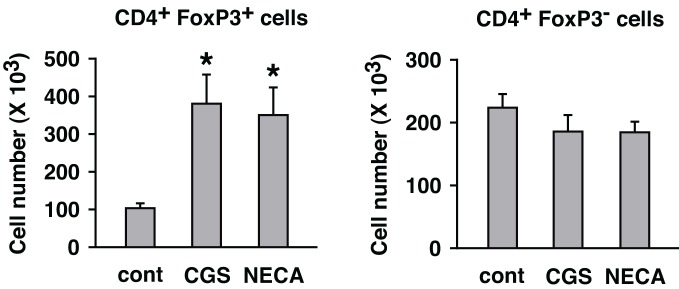
**Numerical increase of Treg after T cell activation in the presence of A2AR agonists.** MLC was set up in the absence of CD8^+^ cells. Two days after restimulation (7 days after initial stimulation), total live cells were enumerated under a microscope. Numbers of H-2K^b+^ CD4^+^ FoxP3^+^ and H-2K^b+^ CD4^+^ FoxP3^−^ cells were estimated from the result of flowcytometric analysis. The number of H-2K^b+^ CD4^+^ cells in the beginning of MLC was approximately 1 × 10^6^ cells including approximately 1 × 10^5^ H-2K^b+^ CD4^+^ FoxP3^+^ cells. Data represent average ± SD of three separate experiments. ^*^P < 0.01 vs. control MLC.

A2AR stimulation enhanced not only regulatory activity of Treg but also the number of Treg. While flowcytometric analysis showed the increased *proportion* of Treg in cultures treated with A2AR agonist, it does not necessarily indicate a *numerical* increase of Treg, especially because A2AR agonists can suppress activation of effector T cells. Total cell number in the culture was counted, and the numbers of CD4^+^ FoxP3^+^ and CD4^+^ FoxP3^−^ cells were calculated from their proportions in the flowcytometric analysis. The result showed a massive increase of CD4^+^ FoxP3^+^ cells and a statistically insignificant decrease of CD4^+^ FoxP3^−^ cells by A2AR agonists (Figure 6).

**Figure 7:**
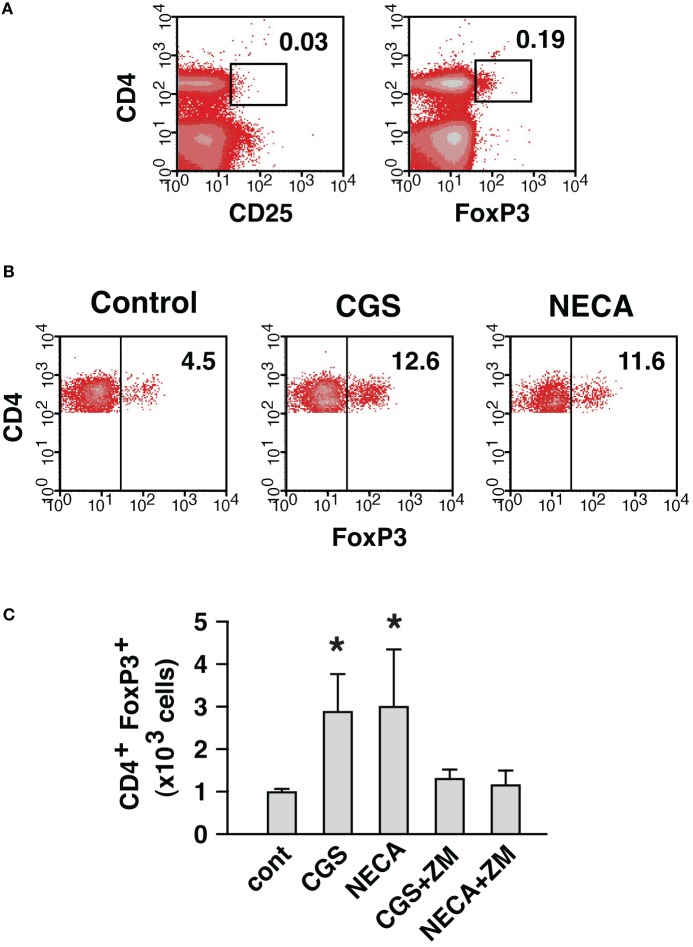
**A2AR stimulation can enhance the induction of CD4^+^ FoxP3^+^ cells from CD4^+^ CD25^−^ cells. (A)** MLC responder cells after the depletion of CD25^+^ cells. The numbers indicate percentages of CD4^+^ CD25^+^ and CD4^+^ FoxP3^+^ cells. The percentage of CD4^+^ cells was approximately 30% because of co-depletion of CD8^+^ cells. **(B)** Induction of CD4^+^ FoxP3^+^ cells from the cells in **(A)**. After 5 (primary stimulation) plus 2 (restimulation) days of MLC, the increase of CD4^+^ FoxP3^+^ cells in control was further enhanced in the presence of A2AR agonists. The data were gated for CD4^+^ cells. The numbers represent percentages of FoxP3^+^ cells in CD4^+^ population. The data shown here represent three separate experiments with similar results. **(C)** Numerical increase of CD4^+^ FoxP3^+^ cells derived from CD4^+^ CD25^−^ cells. Total live cells were enumerated after 7 days, and numbers of H-2K^b+^ CD4^+^ FoxP3^+^ cells were calculated as in Figure 6. Data represent average ± SD of three separate experiments. ^*^P < 0.05 vs. control.

Thus, A2AR stimulation was found to enhance immunoregulation by Treg in both qualitative and quantitative means. The quantitative change, a numerical increase of Treg, could result from the proliferation of preexisting natural Treg (nTreg) and/or the induction of new Treg. A2AR agonist was previously shown to upregulate FoxP3 mRNA in activated T cells (Zarek et al., 2008). Therefore, to monitor the appearance of new Treg, we started MLC with responder cells depleted of nTreg. The depletion of CD25^+^ cells got rid of most FoxP3^+^ cells from the culture (Figure 7A). Some CD25^−^ CD4^+^ FoxP3^+^ cells remained in the culture, but these cells accounted for only 0.5–0.6% of CD4^+^ cells. Control MLC using such responder cells resulted in the induction of CD4^+^ FoxP3^+^ cells to 4.5% of CD4^+^ cells (Figure 7B). A2AR agonists gave rise to further induction of CD4^+^ FoxP3^+^ cells to approximately 12% of CD4^+^ cells. It was also confirmed that the increase of CD4^+^ FoxP3^+^ proportion accompanied significant increase in the absolute number of CD4^+^ FoxP3^+^ cells (Figure 7C). This result suggested that Treg could be derived from CD4^+^ CD25^−^ cells during MLC, and A2AR stimulation promoted this increase.

**Figure 8:**
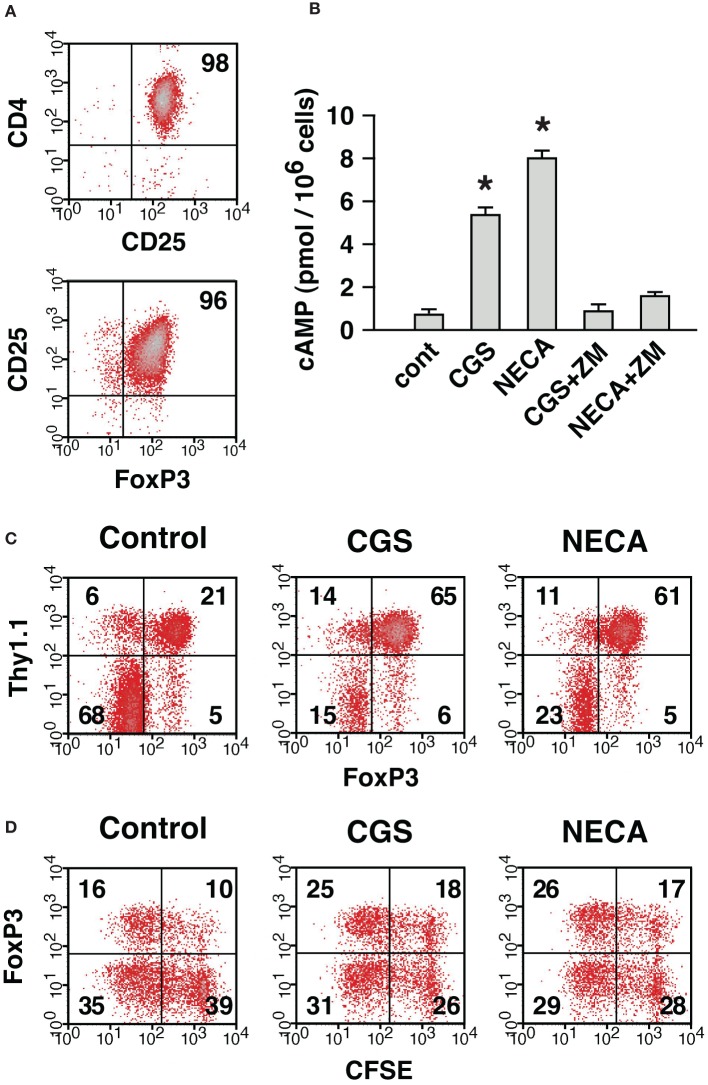
**The expansion of Treg in the presence of A2AR agonist was mostly due to the proliferation of preexisting nTreg. (A)** Purified CD4^+^ CD25^+^ cells represent nTreg. The numbers indicate percentages of CD4^+^ CD25^+^ and CD25^+^ FoxP3^+^ population in the purified cells. These CD4^+^ CD25^+^ cells were obtained from Thy1.1-expressing mice and mixed with Thy1.2-expressing MLC responder cells depleted of CD4^+^ CD25^+^ cells, which were prepared as described for Figure 7A. Thus, MLC responder cells were reconstituted so that only nTreg were expressing Thy1.1. **(B)** Treg express functional A2AR. Incubation of purified CD4^+^ CD25^+^ cells with A2AR agonist induced cAMP production, which was blocked by ZM, A2AR antagonist. Data represent average ± SD of 3 samples. ^*^P < 0.001 vs. control. **(C)** A large part of A2AR-mediated increase of Treg was derived from nTreg. On day 7, the MLC was analyzed for the expression of FoxP3. The data were gated for CD4^+^ cells. The numbers represent percentages of each quadrant. Thy1.1^+^ (upper quadrants) and Thy1.1^−^ (lower quadrants) cells were mostly CD4^+^ CD25^+^ nTreg and CD4^+^ CD25^−^ non-Treg cells in the beginning of culture, respectively. **(D)** Active proliferation of nTreg in MLC and well-maintained FoxP3 expression by treatment with A2AR agonist. MLC responders were reconstituted as in **(C)** except that CD4^+^ CD25^+^ cells from Thy1.1-expressing mice were labeled with CFSE. The panels show CFSE fluorescence and FoxP3 expression in Thy1.1^+^ cells on day 4. The data shown here represent three separate experiments with similar results.

Next, we compared contribution from nTreg and CD4^+^ CD25^−^ cell-derived Treg in the A2AR-mediated increase of CD4^+^ FoxP3^+^ cells. To distinguish preexisting nTreg from CD4^+^ CD25^−^ cell-derived Treg produced during culture, CD4^+^ CD25^+^ cells were purified from Thy1.1^+^ mice. In the purified cells, CD4^+^ CD25^+^ cells were 95–98% and FoxP3-expressing cells were up to 96% of the purified cells (Figure 8A). Purified Treg produced cAMP in response to CGS and NECA, and the increase was blocked by the addition of ZM suggesting functional expression of A2AR on Treg (Figure 8B). These Thy1.1-expressing nTreg were added to CD8^+^, CD25^+^-depleted Thy1.2^+^ spleen cells, which were prepared as in Figure 7A, in order to reconstitute MLC responders. MLC of these responder cells in normal condition yielded CD4^+^ cells predominant with FoxP3^−^ effectors (Figure 8C). These CD4^+^ FoxP3^−^ cells were mostly from Thy1.1^−^ cells as expected, while most of CD4^+^ FoxP3^+^ cells were Thy1.1^+^. There were also some Thy1.1^−^ CD4^+^ FoxP3^+^ cells, but these accounted for only a minor portion (20%) of total CD4^+^ FoxP3^+^ cells (Figure 8C). Treatment with A2AR agonists strongly reduced proportion of FoxP3^−^ effectors and increased CD4^+^ FoxP3^+^ cells. CD4^+^ CD25^−^ cell-derived Treg emerged from Thy1.1^−^ cells were found to increase by A2AR stimulation (FoxP3^+^ within Thy1.1^−^ cells: 7% in control, 29% in CGS and 16% in NECA); however, induction of iTreg had a limited contribution to the increase of total CD4^+^ FoxP3^+^ cells (Figure 8C). The CD4^+^ FoxP3^+^ population after A2AR stimulation was again mostly Thy1.1^+^ cells, which accounted for almost 90% of total CD4^+^ FoxP3^+^ cells. These data suggest that A2AR stimulation may promote CD4^+^ CD25^−^ cell-derived Treg and expansion of nTreg, but the latter mechanism may play a major role in the numerical increase of Treg.

We further analyzed proliferation of nTreg using CFSE-labeled Thy1.1^+^ CD4^+^ CD25^+^ cells. In the reconstituted MLC, nTreg were found to enter massive proliferation (Figure 8D). CD4^+^ CD25^+^ cells proliferated well even in the presence of CGS and NECA, but these A2AR agonists did not further promote proliferation. Interestingly, a large proportion of nTreg lost FoxP3 expression in control MLC, while nTreg with A2AR agonists maintained FoxP3 expression better (Figure 8D). These results suggest that A2AR stimulation can, at least in part, increase the number of Treg by preventing down-regulation of FoxP3.

## Discussion

Adenine, and by extension adenosine, may have been one of the oldest organic compounds on the earth whose appearance preceded the first life form by many 100 million years according to interpretation of life origin experiments (Miller, 1953; Miller and Urey, 1959; Oró and Kimball, 1961). Maybe utilization of adenosine belongs to the oldest and most ancient group of mechanisms regulating immune system in organisms.

Immune cells express A2AR at high levels, and stimulation of A2AR strongly suppresses various immune functions (Ohta and Sitkovsky, 2001; Lukashev et al., 2004; Sitkovsky et al., 2004; Thiel et al., 2005; Belikoff et al., 2011). Interaction of A2AR with endogenously produced adenosine serves as a self-limiting mechanism of inflammation. Immunosuppression through the adenosine-A2AR pathway seems to be critical in maintaining inflammation to proper levels. This is because the lack of A2AR led to exaggeration of proinflammatory responses and subsequent inflammatory tissue damage (Ohta and Sitkovsky, 2001; Thiel et al., 2005; Ohta et al., 2006).

Activation of CD4^+^ and CD8^+^ T cells is under control of the adenosine-A2AR pathway. Our previous results have shown A2AR-mediated inhibition of T cell activities such as proliferation, cytokines production and cytotoxicity (Huang et al., 1997; Ohta et al., 2009). This inhibitory effect of adenosine and its analogs is based on a direct action via A2AR expressed on T cells. It is consistent with the interruption of T cell receptor signaling by cAMP increase after A2AR stimulation (Vang et al., 2001; Linnemann et al., 2009). In addition to the inhibitory effect on T cell priming, A2AR stimulation produced activated T cells with impaired effector function. Indeed, T cells activated in the presence of A2AR agonist showed persistently lower cytokine-producing activity even after the removal of A2AR agonist (Zarek et al., 2008; Ohta et al., 2009).

In our studies on CTL development, we noticed the uniqueness of MLC, where massive expansion of Teff and increase of Treg were observed in the same culture. This may mimic immune responses *in vivo*: i.e., promotion of proinflammatory activities and compensatory anti-inflammatory responses to prevent excessive tissue destruction. Such responses were not observed in artificial stimulation of T cells with anti-CD3 mAb as Teff strongly overwhelm the culture. The current study revealed that A2AR stimulation inhibited activation of effector T cells and, at the same time, increased the number of Treg (Figures 1, 2, and 6). CD4^+^ FoxP3^+^ cells from the MLC system express CD25, CTLA-4, CD39, and CD73 at high levels and have immunoregulatory activities as it has known for Treg induced by other methods (Figures 3–5). Our data suggests that A2AR not only directly inhibits T cell activation but also produces immunosuppressive cellular environment by inducing massive increase of Treg. Therefore, immunosuppressive concentration of extracellular adenosine may provide a long-lasting immunoregulatory effect even after the disappearance of adenosine.

The A2AR-mediated numerical change of Treg was due to increase of both nTreg and CD4^+^ CD25^−^ cell-derived Treg. MLC after the depletion of nTreg showed development of Treg from CD4^+^ CD25^−^ cells and its enhancement by A2AR stimulation (Figure 7). The increase of CD4^+^ CD25^−^ cell-derived Treg in normal MLC, in the presence of nTreg, required distinction for the origin of CD4^+^ FoxP3^+^ cells in the product. Reconstitution of MLC responders using Thy1.1-expressing nTreg made this distinction possible, and the A2AR-mediated increase of CD4^+^ CD25^−^ cell-derived Treg was confirmed in regular MLC (Figure 8). The increase of new Treg is consistent with a previous paper reporting mRNA upregulation of FoxP3 and LAG-3 in CGS-treated T cell culture (Zarek et al., 2008). Although the increase of Treg from CD4^+^ CD25^−^ cells was significant, these Treg accounted for only a minor portion of A2AR-mediated increase of Treg. Most of Treg after MLC was found to derive from nTreg (Figure 8). Purified nTreg was reported to proliferate when cultured with allogenic dendritic cells and IL-2 (Yamazaki et al., 2006). IL-2 was not added in our culture system, but activated CD4^+^ cells might have produced IL-2 and supported proliferation of Treg. Indeed, massive proliferation of nTreg was observed in the current study (Figure 8D). Other possible reasons for A2AR-mediated enhancement of nTreg include prevention of FoxP3 down-regulation and cell death, which have been observed during Treg culture (Strauss et al., 2007; Hoffmann et al., 2009). In the current study, a number of nTreg were found to lose their FoxP3 expression after activation, while A2AR stimulation considerably prevented FoxP3 loss (Figure 8D). This mechanism may be partly responsible for the increase of CD4^+^ FoxP3^+^ cells treated with A2AR agonists. In addition, A2AR agonists are known to block activation-induced cell death of CD4^+^ T cells (Himer et al., 2010).

Not only A2AR agonist increased the number of Treg, it also enhanced their immunoregulatory activity. T cell suppression assay showed that the same number of Treg from CGS/NECA-treated MLC had 4-times stronger regulatory activity than that from control MLC (Figure 3). The mechanism how Treg suppress T cell activation is still unclear, but CTLA-4 and CD25 represent important candidates. Pathogenesis of autoimmune disorders in mice with Treg-specific CTLA-4 deficiency demonstrated the importance of CTLA-4 in their regulatory activity (Wing et al., 2008; Sakaguchi et al., 2009). CD25 expression on Treg at high levels is considered to withdraw IL-2 from the microenvironment and induce effector cell death because of IL-2 deprivation (Pandiyan et al., 2007, 2011). The A2AR-mediated upregulation of CTLA-4 and CD25 on Treg (Figures 1–3) may support the enhancement of regulatory activity.

We assumed that it would be very effective immunosuppression if the activities of Treg were additive to or synergistic with other immunosuppressive mechanisms in the microenvironment. Accordingly, it was hypothesized that the immunosuppressive cytokines and molecules such as CTLA-4 would be increased on Treg by the same mechanism that mediates the hypoxia-adenosinergic immunosuppression (Sitkovsky, 2009). The detailed studies of hypoxia response element (HRE)/hypoxia-inducible factor-1α (HIF-1α) and cAMP response element (CRE)/cAMP response element binding protein (CREB) have been implicated in Treg activities only by circumstantial set of data (Sitkovsky, 2009) and the direct studies of CRE and HRE in Treg are still to be performed.

Tumors contain high levels of extracellular adenosine (Blay et al., 1997; Ohta et al., 2006). Adenosine in tumor microenvironment may be one of the important immunosuppressive mechanisms, which discourage anti-tumor immune responses, because A2AR-deficient mice could efficiently eradicate tumors, while wild-type mice could not (Ohta et al., 2006). One important reason for adenosine-mediated inactivation of anti-tumor immune responses would be a direct action at A2AR on T cells. However, the present data also suggest that the adenosine-A2AR signaling may enhance Treg in tumors. Tumors contain overwhelming number of Treg to suppress effector T cells (Turk et al., 2004; Antony et al., 2005; Sitkovsky et al., 2008; Facciabene et al., 2011). There might be a contribution from intratumoral high concentration of adenosine to the increase of Treg. Moreover, A2AR stimulation may enhance the regulatory activity of Treg in tumors and further inactivate anti-tumor immune responses. Physiological control of Treg activity *in vivo* by extracellular adenosine is yet to be determined.

While Treg is a target to be discouraged for the improvement of tumor immunotherapy, transfusion of Treg is a promising approach for the treatment of autoimmune diseases and allogenic reaction after transplantation (Riley et al., 2009; Matsuoka et al., 2010). Because large doses of Treg are necessary to suppress GVHD, Treg require massive expansion *ex vivo* before transfer, but the expansion of Treg is somewhat challenging. It is difficult to start the expansion from a large number of Treg because of low frequency of Treg in peripheral blood. In addition, since both Treg and activated effector T cells share CD4^+^ CD25^+^ phenotype, polyclonal activation of T cell could result in considerable contamination by effector T cells (Riley et al., 2009). Treatment with A2AR agonist induces expansion of Treg, while it suppresses activation of effector T cells. Such a culture condition favoring Treg outgrowth may be suitable for the expansion of Treg. Effects of A2AR agonists on human Treg will need to be examined for numerical increase and enhancement of regulatory activity. Dependent on the nature of human Treg, optimization of culture condition is expected to improve the recovery of functionally enhanced Treg.

In conclusion, we found that T cell activation in the presence of A2AR agonist resulted in expansion of Treg. After the A2AR-mediated expansion, Treg acquired stronger immunoregulatory activity. The quantitative and qualitative enhancement of Treg by the adenosine-A2AR pathway may be relevant to the establishment of longer-lasting immunomodulation. This mechanism may be utilized in the expansion of Treg for treatment of autoimmune diseases and GVHD.

### Conflict of interest statement

The authors declare that the research was conducted in the absence of any commercial or financial relationships that could be construed as a potential conflict of interest.
